# Individual random effects model for differences in trait distribution among respondents

**DOI:** 10.1038/s41598-024-62479-0

**Published:** 2024-05-25

**Authors:** Rui Wu, Xuliang Gao, Shiquan Pan, Fan Wang, Shouying Zhao

**Affiliations:** 1 School of Psychology, Guizhou Normal University, Guiyang, China; 2 College of Humanities and Management, Guizhou University of Traditional Chinese Medicine, Guiyang, China; 3 School of Humanities, Guizhou Medical University, Guiyang, China; 4 Kaili University, Kaili, China

**Keywords:** Psychology, Human behaviour

## Abstract

The homogeneity hypothesis is a common assumption in classic measurement. However, the item response theory model assumes that different respondents with same ability have the same option probabilities, which may not hold. The aim of this study is to propose a new individual random effect model that accounts for the differences in option probabilities among respondents with same latent traits by using within-person variance. The performance of the new model is evaluated through simulation studies and real data using the PRESUPP scale of PISA. The model parameters are estimated by the MCMC method. The results show that the individual random effect model can provide more accurate parameter estimates and obtain a scale parameter to describe the distribution of respondents’ abilities, under different within-person variances. The new model has lower RMSE and better model fit than the classic IRT model.

## Introduction

Researchers in psychology, education and other social sciences often emphasize "latent traits", which cannot be read directly off a numerical scale. For this case, there is a lack of tangible and stable tools for the measurement of these latent traits (hereafter referred to as abilities). We can only design a set of items to assess or estimate the abilities indirectly through the respondents’ responses to the items^1^. To investigate the relationship between items and abilities of respondents, item response theory (IRT), proposed by Lord^2^, was formally developed.

According to the classic IRT models and their basic assumptions, the option probabilities to a single item are only related to the respondents’ abilities, which is usually defined by $\theta$. Respondents with the same $\theta$ have the same option probabilities on each item. In practice, however, this principle may very well be violated. For example, the study of differential item functioning found that there may be systematic differences in item parameters among different groups of respondents and even within groups. Sometimes, such differences indicate that some secondary factors are measured in the items. However, in most cases, we do not know the underlying causes of differential item functioning^3^. Studies on response style show that the option probabilities are influenced by personal characteristics irrelevant to the measurement target, and respondents may have preference for selecting certain options. This difference is hard to account for by a new dimension^4–10^.

To account for this difference, researchers have proposed various models. For example, Everitt^11^ and Titterington^12^ proposed the discrete mixed distribution model, assuming that the observed data came from the mixture of two or more potential populations. Based on this, the random item effects model is proposed, which implied that a particular IRT model is not suitable to all the respondents, the parameter sets (difficult parameters, slope parameters, etc.) of items could then vary across subgroups^13^. The finite mixing model^14^ believes that the respondents have different cultures or backgrounds, posited the existence of a discrete metric space and allowing heterogeneity among different metric spaces. The bifactor model and higher-order model focus on factor levels, assuming that there is a global factor that can explain the common variation of all items. The difference in global factors is used to explain the differences among respondents other than the ability. The difference is that the bifactor model believes that the global factor has only a direct effect on the observed variables, and the higher-order model is based on a complete mediator^15^, which means that higher-order factors completely affected the observed variables through lower-order factors. The interaction model introduced a random interaction variable, the pairing of item* i* and respondent* j* produced a new interaction variable ${\epsilon }_{ij}$, and the differences in parameters among different respondent-item combinations were due to the differences in the interaction variables^16^. The study of response styles in self-report rating-scale instruments assume selection among response categories is often simultaneously influenced by both substantive and response-style traits^17^. The latent space item response model assumed that both items and respondents were embedded in an unobserved metric space, with the probability of a correct response decreasing as a function of the distance between the respondent’s and the item’s position in the latent space^18^. Although these models have been successfully applied in practice, they were not free of limitations. For example, the random item effect model and finite mixture model required the background information of the respondents to be known before data analysis and needed to ensure homogeneity within the subgroups. The bifactor model and the higher-order factor model introduced at least one additional factor to explain the responses pattern of the respondents, and it did not work in the unidimensional condition. The response styles model required more than two options of each item. The interaction model and latent space model, which were the most flexible, respectively proposed interaction variables and latent space distance to describe the interaction between the respondents and the items, but these two parameters were relative measurements. They expressed the relationship between a specific respondent and a specific item, and thus, they were suitable for the interpretation of the secondary factors. In practice, sometimes we cannot find and define secondary factors, even though some scales do not involve secondary factors. The two-dimensional latent space model had the problem of insufficient explanatory power when there were too many items and respondents, while the multidimensional latent space model was not easy to calculate and understand.

Personality researchers were initially interested in differences of within-person variance and measure it by repeatedly administering the same items^19–21^. Recently, based on their research, Williams^22^ proposed the Bayesian nonlinear mixed effects location-scale model (NL-MELSM). This model allows within-person variance to follow a nonlinear trajectory in learning, which can determine whether variability is reduced during learning. Lin^23^ proposed a multiple sharing parameter model, where the longitudinal outcomes of multiple densities are modeled by the mixed-effect location-scale model and further linked to the corresponding deletion mechanism through the shared respondent random effect. However, it cannot be ignored that previous exposure to test will influence the performance on the test. Even if retesting were done under identical conditions, the examinee is no longer the same person in the sense that relevant experiences have occurred that had not occurred before the first testing^24^.

Williams et al.^25^ proposed a perspective that went beyond homogeneous variance and viewed modeling within-person variance as an opportunity to gain a richer understanding of psychological processes. In this framework, a new model was introduced, where within-person variance is considered as a factor affecting respondents' option probabilities. The within-person variances are named individual random effects, which represents the magnitude of within-person variance of the respondents' ability, which is helpful to obtain the distribution characteristics of the respondents' ability.

The development of the new model is inspired by the generalizability theory (GT) and the research on within-person variance in psychological measurement. According to the GT, all measurements have variances, which may arise from the measurement tools, and the users of the tools not mastering the essentials, while the measurement conditions, environments or the respondents do not cooperate. In short, there are various sources for measurement variance, include within and between persons.

On the other hand, some researchers have argued that individual internal variation is not only inevitable, but also meaningful, from the perspectives of both psychological mechanisms and mathematical statistics^26–28^. All the measurement variances are not meaningless, it is the nonnegative parameter included in the complete measurement result, which reasonably gives the measured value with dispersion. Omission or repeated consideration of variance sources result in reflecting incorrect measurement results of the actual measurement status and affect the validity and reliability of measurement^29^. Classic measurement tends to assume that the respondents are homogeneous within the group, and the variance stems from the limitation of measurement tools. In the framework of the mixed-effect location-scale model, the goal of psychological measurement involves not only the measurement of location (or mean) but also the measurement of scale (or within-person variance). Within-person variance is considered not only to reflect measurement error but also to reflect system information^30^.

Ferrando^31^ proposed the same model based on Thurstone scaling, but eventually simplified it for the ease of parameter estimation, and used a two-stage parameter estimation method that may introduce some errors.

In this study, we introduced the individual random effect model (IREM) by combining the mixed-effect location-scale model and the IRT. The within-person variance in the model is incorporated into the IRT model as the respondents’ parameter. The differences in option probabilities among respondents are regarded as the result of different within-person variances. This change in perspective comes with important benefits, such as: (a) We work with the original item response data rather than functions of item response data, (b) we estimate within-person variance as the respondents’ parameter, rather than integrating it into item parameters, which facilitates a richer understanding of psychological processes, © we use the data of one measurement, not longitudinal data, for parameter estimation to avoid within-person variance being confounded with the change of the mean, (d) our approach is closely related to the IRT model, which facilitates interpretation.

To demonstrate the advantages of the model, we derive the IRT model and the variance decomposition principle, and then compare it with the classic IRT model through simulation and real data studies. In conclusion, the model in this study introduces a new scale parameter and has certain benefits in the estimated accuracy of $\theta$. It is expected to offer a new perspective for studying the different option probabilities among respondents in the IRT model.

## Model

### Normal ogive model

In 1952, Lord proposed the first IRT model, the two-parameter normal ogive curve model, and applied this model to the measurement of academic achievement and attitude^2^. It included three basic assumptions: (a) unidimensional, (b) local independence, and (c) the formal hypothesis of the item characteristic curve, namely, the monotone increasing hypothesis, which is given by the basic equation:

1 $$P\left(X = 1|\theta , b\right) = \frac{1}{\sqrt{2\pi }}{\int }_{-\infty }^{a\left(\theta - b\right)}{e}^{-\frac{{t}^{2}}{2}}dt,$$

where $b$ is the difficulty parameter, $a$ is the slope parameter for the item, and $\theta$ represents the respondent’s ability; it represents the area under the standardized normal curve of the Z score from $-\infty$ to $a\left(\theta - b\right)$.

### Mathematical derivation of the individual random effect model

The individual random effects model can be derived based on the mathematical foundation of the normal ogive model. Suppose respondent* i* has ability $\theta$ and a binary item *j* has difficulty parameter b. $Y$ is defined as the observed response value (score). There must be a threshold $\eta$, whenever $\theta$ is greater than $\eta$, $Y=1$; otherwise, $Y=0$. The distribution of $\eta$ is normal, with the mean denoted by $b$ and the variance denoted by ${\sigma }^{2}$; thus, the frequency distribution of $\eta$ is given by

2 $$\varphi \left(\eta \right)=\frac{1}{\sqrt{2\pi }\sigma }{e}^{-{ \frac{\left(\eta -b\right)}{2{\sigma }^{2}}}^{2}}.$$

Let $t=\frac{\eta -b}{\sigma }$*,* then $t\sim N\left(\text{0,1}\right)$ and $\eta =t\sigma +b$. The probability that the respondent will score 1 on the item is

$$P\left(Y = 1|\theta ,b\right)=P\left(\theta >\eta |\theta ,b\right)=P\left(\theta >t\sigma +b|\theta ,b\right)=P\left(t<\frac{\theta -b}{\sigma }|\theta ,b\right)$$

3 $$=\frac{1}{\sqrt{2\pi }}{\int }_{-\infty }^{\frac{\theta -b}{\sigma }}{e}^{-\frac{{t}^{2}}{2}}dt.$$

Let $a=\frac{1}{\sigma }$, then it will be the same normal ogive model as (1), which consider $\theta$ to be a fixed value throughout the test and threshold η goes up and down around $b$.

As Williams et al.^25^ proposed, any estimate of an individual ability, $\theta$, was an estimate of an average, and it was assumed that the real ability, ${\theta }^{*}$, was normal, with the mean denoted by $\theta$ and the variance denoted by ${\varepsilon }^{2}$; thus, the frequency distribution of ${\theta }^{*}$ is given by,

4 $$\varphi \left({\theta }^{*}\right)=\frac{1}{\sqrt{2\pi }\varepsilon }{e}^{-{ \frac{\left({\theta }^{*}- \theta \right)}{2{\varepsilon }^{2}}}^{2}}.$$

Then, the probability of respondent *i* endorsing item *j* is given by,

5 $$P\left(Y = 1|\theta ,b\right)=P\left({\theta }^{*}>\eta |\theta ,b\right).$$

Let $z={\theta }^{*}-\eta$ and $t{\prime}=\frac{z-(\theta -b)}{\sqrt{{\varepsilon }^{2}+{\sigma }^{2}}}$, then $z\sim N\left(\theta -b,{\varepsilon }^{2}+{\sigma }^{2}\right)$ and $t\sim N\left(\text{0,1}\right)$ and ${t}{\prime}=z\sqrt{{\varepsilon }^{2}+{\sigma }^{2}}+(\theta -b)$. The probability that the respondent will score 1 on the item is,

6 $$P\left(Y = 1|\theta ,b\right)=P\left({\theta }^{*}>\eta |\theta ,b\right)=P\left({\theta }^{*}-\eta >0|\theta ,b\right)=P\left({\theta }^{*}-\eta -\left(\theta -b\right)>-\left(\theta -b\right)|\theta ,b\right)=P\left(\frac{{\theta }^{*}-\eta -\left(\theta -b\right)}{\sqrt{{\varepsilon }^{2}+{\sigma }^{2}}}>\frac{-\left(\theta -b\right)}{\sqrt{{\varepsilon }^{2}+{\sigma }^{2}}}|\theta ,b\right) =P\left({t}{\prime}>\frac{-\left(\theta -b\right)}{\sqrt{{\varepsilon }^{2}+{\sigma }^{2}}}|\theta ,b\right)=\frac{1}{\sqrt{2\pi }}{\int }_{\frac{-\left(\theta -b\right)}{\sqrt{{\varepsilon }^{2}+{\sigma }^{2}}}}^{\infty }{e}^{-\frac{{t}^{2}}{2}}d{t}{\prime}=\frac{1}{\sqrt{2\pi }}{\int }_{-\infty }^{\frac{\theta -b}{\sqrt{{\varepsilon }^{2}+{\sigma }^{2}}}}{e}^{-\frac{{t}^{2}}{2}}d{t}{\prime}.$$

The integration function of normal ogive model cannot be expressed as elementary function, which is difficult to use in practice. This urges people to look for alternative models, and the logistic model is proposed in this context.

Haley^32^ proved that for $X\in R$, the relationship between logistic model and normal ogive model can be stated as

7 $$\left|\frac{1}{\sqrt{2\pi }}{\int }_{-\infty }^{x}{e}^{-\frac{{t}^{2}}{2}}dt - \frac{1}{1 + {e}^{-1.7x}}\right|<0.01.$$

Therefore, the logistic model can be used as an approximation of the normal ogive model, which is much easier to calculate. Derived from (7) and (8), the new model assumes the following item response function,

8 $$P\left(X = 1|\theta ,b\right) =\frac{1}{1 + {e}^{-D\sqrt{\frac{1}{{\varepsilon }^{2}+{\sigma }^{2}}}\left(\theta - b\right)}}.$$

It is worth noting that when ${\varepsilon }^{2}$ is constant, the individual random effects model is equivalent to the two-parameter IRT model. Thus, the individual random effects model can be regarded as a generalization of the two-parameter model. In practice, we determine whether ${\varepsilon }^{2}$ is constant via model selection, as described in Sect. "Simulation study". The value of the individual random effects model is that it explores the source of differences in the option probabilities of different respondents with the same ability on the same item under the unidimensional model, describing this by the difference of ${\varepsilon }^{2}$.

### Properties

#### Individual random effects model

The individual random effects model described above is derived from the classic IRT model and can be regarded as a special two-parameter mixed model. Specifically, the respondent compares ${\theta }^{*}$ with $\eta$, and when ${\theta }^{*}>\eta$, the respondent obtains a score of “1” for the item. However, there is variance both from the item and the respondent, and $\theta$, $b$, ${\varepsilon }^{2}$ and ${\sigma }^{2}$ jointly affect the relative position of ${\theta }^{*}$ and $\eta$, thereby affecting the respondent's response. Compared with the classic IRT model, the individual random effects model does not require homogeneity in groups. It is worth noting that the respondent group is not always heterogeneous. Identifying heterogeneity and whether the use of individual random effects models in a homogeneous group leads to undesirable results needs to be explored in our research.

There are many similarities between the individual random effects model and the mixed model. They both show that the item parameters are different for different respondents. According to the mixed model, in different subgroups, the same item may have different slope and difficulty parameters, which is caused by the specific culture or background of the subgroups. The purpose of the mixed model is to distinguish the differences between subgroups and estimate the respondents’ parameters more accurately. The individual random effects model focuses on the heterogeneity that may exist between any two respondents to estimate a new parameter and to calculate the different distribution of the respondent’s ability, which is meaningful for predicting individual behavior.

#### Practical advantages

A unique advantage of the proposed individual random effects model is that it provides a parameter used to explain the differences in the option probabilities of respondents with the same ability. Due to different within-person variances, the respondents with the same ability show different option probabilities, which is in line with reality. At the same time, we can effectively obtain the specific distribution of the respondents’ abilities by estimating the relevant variance of the respondents, which is important for improving measurement information and predicting individual behavior.

#### Theoretical advantages

One of the theoretical advantages of the proposed individual random effects model is that it weakens the conditional independence assumption of the classic IRT model and the homogeneity assumption of the classic measurement.

##### Conditional independence assumptions

The proposed individual random effects model is based on the following conditional independence assumption:

$$P\left({\varvec{Y}}=y|{\varvec{\theta}},{\varvec{b}},{\varvec{\sigma}},{\varvec{\varepsilon}}\right)=\prod_{j=1}^{J}\prod_{i=1}^{I}P\left({Y}_{ij}={y}_{ij}|{\theta }_{i},{b}_{j},{\sigma }_{j},{\varepsilon }_{i}\right),$$

where the Y the full response matrix and ${\varvec{\theta}}=\left({\theta }_{1},\dots ,{\theta }_{I}\right),{\varvec{b}}=\left({b}_{1},\dots ,{b}_{J}\right),{\varvec{\sigma}}=\left({\sigma }_{1},\dots ,{\sigma }_{J}\right),{\varvec{\varepsilon}}=\left({\varepsilon }_{1},\dots ,{\varepsilon }_{I}\right)$. In words, the item responses are assumed to be independent conditional on the ability of the respondents, the difficulty of the items, and the variance caused by the respondents and the items. This conditional independence assumption is weaker than the conditional independence of the classic IRT model. The classic IRT model requires the (conditional) distribution of item scores to be independent of each other within any respondent group, and the item scores are only related to the ability $\theta$^33^.

The weaker conditional independence assumption of the individual random effects model allows for differences between respondents or items with the same $\theta$ or $b$.

##### Homogeneity assumptions

The assumption of homogeneity is a prerequisite for classic measurements, including classic IRT. In measurement, homogeneity is manifested in that the respondents of different subgroups are indistinguishable: the subgroups have the same scale, similar knowledge structure and backgrounds, and there is no difference in the overall variance between the different subgroups. The formula can be expressed as ${y}_{ij}={\beta }_{0}+{u}_{0i}+{\epsilon }_{ij}$, where ${\beta }_{0}$ is the fixed effect and ${u}_{0i}$ is the individual deviation. ${\epsilon }_{ij}$ are residuals. Under the condition of homogeneity, they are assumed to be normal distributions with constant variance, i.e. ${\epsilon }_{ij}\sim normal\left(0, \sigma \right)$.

This is also the basis for the same option probabilities of respondents with the same ability. In measurement, homogeneity cannot be strictly guaranteed. Individual random effects models can estimate within-person variance. We are not treating homogeneous variance as a hypothesis that needs to be satisfied or treating within-person variance as noise. Rather, within-person variance is considered as a factor that affects the respondent's option probabilities and is included in the estimate.

## Parameter estimate

When estimating the parameters of the complex probability density equation, the MCMC method is easier than other methods. Therefore, we use the MCMC method to estimate the parameters of the individual random effects model and implement the method based on the RSTAN.

Referring to previous studies^34^, we use the following priors:

$${\theta }_{i}\sim normal\left(0, 1\right), i = 1, \dots , N$$

$$\mathit{ln}{a}_{j}\sim normal\left(0, {{\sigma }_{a}}^{2}\right),j = 1, \dots , K$$

$${b}_{j}\sim normal\left({\mu }_{b}, {{\sigma }_{b}}^{2}\right), j = 1, \dots , K$$

$${{\mu }_{b}}_{j}\sim cauchy (0, 5), j = 1, \dots , K$$

$${{\sigma }_{b}}_{j}\sim cauchy (0, 5), {{\sigma }_{b}}_{j}>0, j = 1, \dots , K$$

$${{\sigma }_{a}}_{j}\sim cauchy\left(0, 5\right), {{\sigma }_{a}}_{j}>0, j = 1, \dots , K$$

For the individual random effects model, without loss of generality, we stipulate its relevant priors as follows:

$$\text{ln}{\sigma }_{j}\sim normal\left(0,1\right), j = 1, \dots ,\text{ K}$$

$$\text{ln}{\varepsilon }_{i}\sim normal\left(0,1\right), i = 1, \dots ,\text{ N}$$

To guarantee $\widehat{R}$<1.2 for all parameter estimates^35^, the MCMC ran included 10,000 iterations, with the first 5,000 iterations discarded as a burn-in period. The MCMC process was implemented with Stan software, and simulation algorithm was written in R. The entire process ran on a computer with Intel(R) Core (TM) i7-11700 K CPU and 64G RAM.

### Identifiability

The log odds of a correct response is invariant to translations, reflections, and rotations of the positions of respondents and items, because the log odds depends on the positions through the distances, and the distances are invariant under the said transformations. Consequently, the likelihood function is invariant under the same transformations. The same form of identifiability issue arises in random effect models. Such identifiability issues can be resolved by post-processing the MCMC output with Procrustes matching^36^. However, the results need to be interpreted with care, because there are many random effect configurations that give rise to the same distances. So, the estimated random effects should be interpreted in terms of relative size, not in terms of actual values.

## Simulation study

To compare the individual random effects model and the classic IRT models, Monte Carlo simulations are used. Compared with the classic model, the individual random effects model mainly incorporates the within-person variance of the respondents. The main factors that have substantial impacts on the accuracy of parameter estimate are the length of the test and the number of respondents. Therefore, this experiment contains 3 independent variables: (a) sample size (200, 500, 1000), (b) test length (20, 30, 50), and (c) the scale of within-person variance (${\sigma }_{P}$ is constant or log-normal distribution, which is the classic two-parameter model and new model). To reduce random errors, the simulation is repeated 30 times under each condition, and the results are averaged.

### Data generation

#### Generation of respondents’ parameters

The number of respondents contains three levels: N = 200, 500, 1000. The within-person variances of respondents have two levels: the within-person variances are different where $\text{ln}\varepsilon \sim normal(0, 1)$ and the within-person variances are constant where $\varepsilon \equiv 1$.

#### Generation of items’ parameters

The number of items contain three levels, K = 20, 30, and 50. The difficulty of the items is generated according to $b\sim normal\left(0, 1\right)$, and the slope of the 2PL model item is generated according to $\text{ln}\sigma \sim normal\left(0, 1\right)$.

### Data analysis

To assess the model's estimate accuracy, the following two indicators are used to measure the model's estimate accuracy of the tested ability parameters:

(1) The root mean square error:

$$RMSE=\sqrt{\frac{{\sum }_{r=1}^{N}{\left(\widehat{\theta }-\theta \right)}^{2}}{N}.}$$

(2) The coefficient of deviation:

$$Bias=\frac{1}{N}\sum_{I=1}^{N}\left(\widehat{\theta }-\theta \right),$$

where $\widehat{\theta }$ is the estimate of mean ability, $\theta$ is the real mean ability, and N is the number of respondents.

(3) $$\widehat{\varepsilon }$$’s standard deviation $${S}_{\widehat{\varepsilon }}$$:

In practice, for a given dataset, it is natural to consider whether it is a classic IRT model with a constant $\varepsilon$ or an individual random effects model with a variable $\varepsilon$. If the data is generated by an individual random effects model, then ${S}_{\widehat{\varepsilon }}$ is greater than zero and the data analysis for the respondents and items should be based on the individual random effects model with the variable $\varepsilon$ parameter, otherwise, the classic IRT model is sufficient. The calculation is as follows:

$${S}_{\widehat{\varepsilon }}=\sqrt{\sum_{i=1}^{N}\frac{{\left({\widehat{\varepsilon }}_{i}-{\mu }_{\widehat{\varepsilon }}\right)}^{2}}{N}}.$$

(4) Correlation coefficient of $\varepsilon$ and $\widehat{\varepsilon }$:

$\widehat{\varepsilon }$ is the parameter describing the size of the within-person variance. Therefore, it is worth exploring whether it can be estimated effectively. We use the correlation coefficient of $\varepsilon$ and $\widehat{\varepsilon }$ to describe the validity of the estimates.

### Results

**Figure 1:**
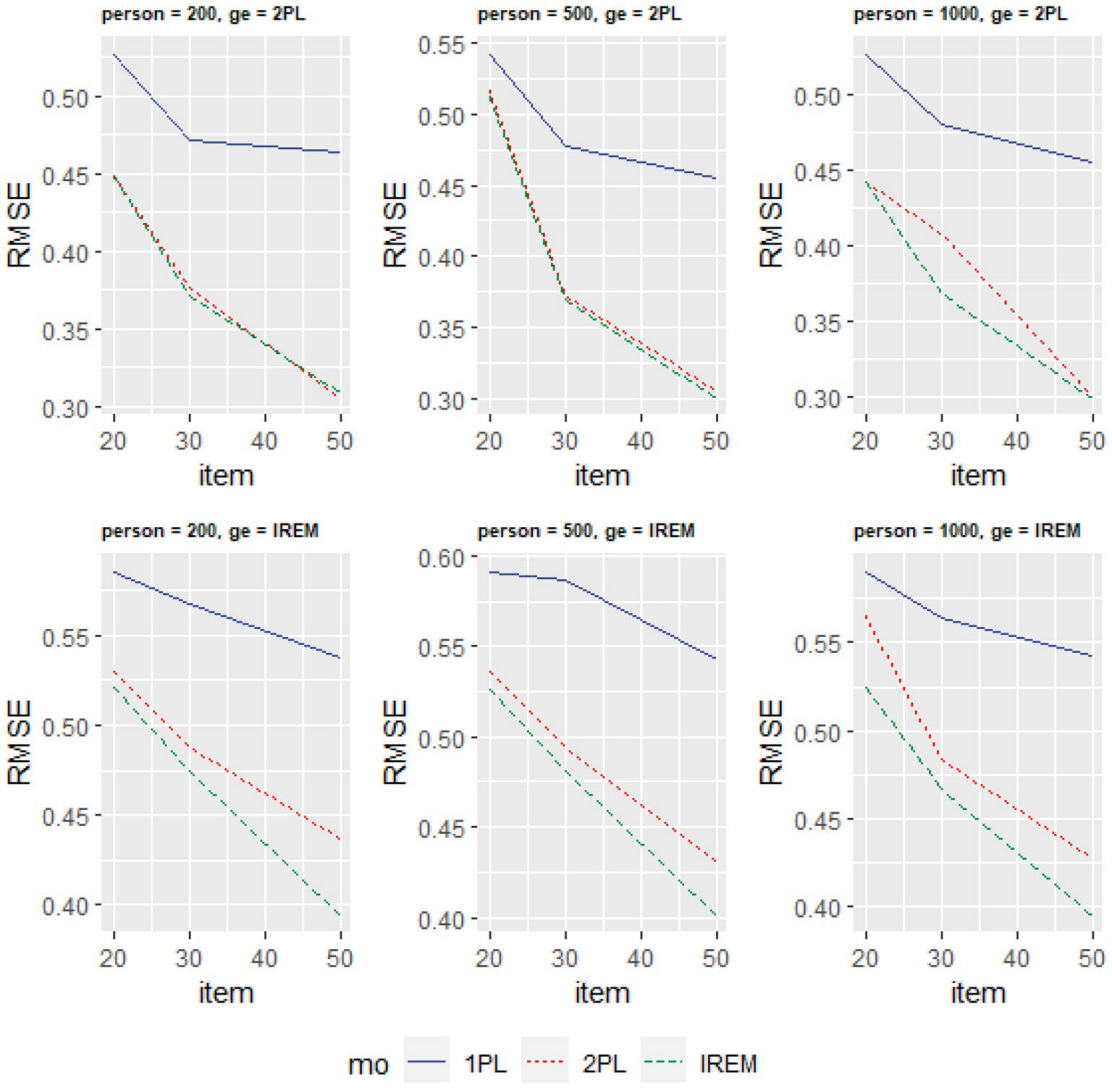
Comparison of RMSE of three models under different conditions.

Table 1 and Fig. 1 show the RMSE under different conditions.

**Table 1:** RMSE values of potential trait levels of respondents under various conditions generated by 2PL.

Respondents	Items	1PL	2PL	IREM
200	20	0.527	0.449	0.448
	30	0.472	0.377	0.371
	50	0.464	0.305	0.308
500	20	0.542	0.516	0.512
	30	0.477	0.372	0.370
	50	0.455	0.305	0.300
1000	20	0.526	0.442	0.442
	30	0.480	0.407	0.368
	50	0.455	0.300	0.299

The response data is generated based without any differences in within-person variances and differences in within-person variances (that is, the 2PL model and the IREM). The results in Table 1 show that for the data generated by 2PL, regardless of how the test length and sample size change, the RMSE of parameter estimate using the new model is considerably smaller than that of the 1PL and similar to that of the 2PL. The results in Table 2 show that when the data is generated by the new model, the RMSE of parameter estimate using the new model is considerably smaller than 1PL and 2PL, and the test length has an important influence on the parameter recovery. As the length of the test increases, the new model has a more substantial downward trend than 1PL and 2PL, which shows that the IREM can provide more robust and accurate estimates (see Fig. 1). It is worth noting that, under all conditions, the RMSE is greater than 0.3, which is related to the larger random variation of the simulation setting, because ${a}_{ij}=\frac{1}{{\sigma }_{ij}}=\sqrt{\frac{1}{{{\sigma }_{I}^{2}}_{i}+{{\sigma }_{P}^{2}}_{j}}}$, when the item variance and the respondent within-person variance mean is 1, the average slope is approximately 0.71, and the amount of item information is small. When the length of the test is limited, the standard error of the test is large^37^.

**Table 2:** RMSE values of potential trait levels of respondents under various conditions generated by the new model.

Number of Respondents	Items	1PL	2PL	IREM
200	20	0.585	0.530	0.521
	30	0.567	0.488	0.474
	50	0.537	0.437	0.394
500	20	0.591	0.536	0.526
	30	0.587	0.494	0.481
	50	0.543	0.431	0.401
1000	20	0.590	0.565	0.524
	30	0.564	0.484	0.467
	50	0.543	0.428	0.395

Under all conditions, the bias value is close to 0 (less than 0.05), indicating that regardless of whether the 2PL model or the IREM is used to generate the response matrix, the point estimates of all models are unbiased estimates of the respondent’s ability.

**Figure 2:**
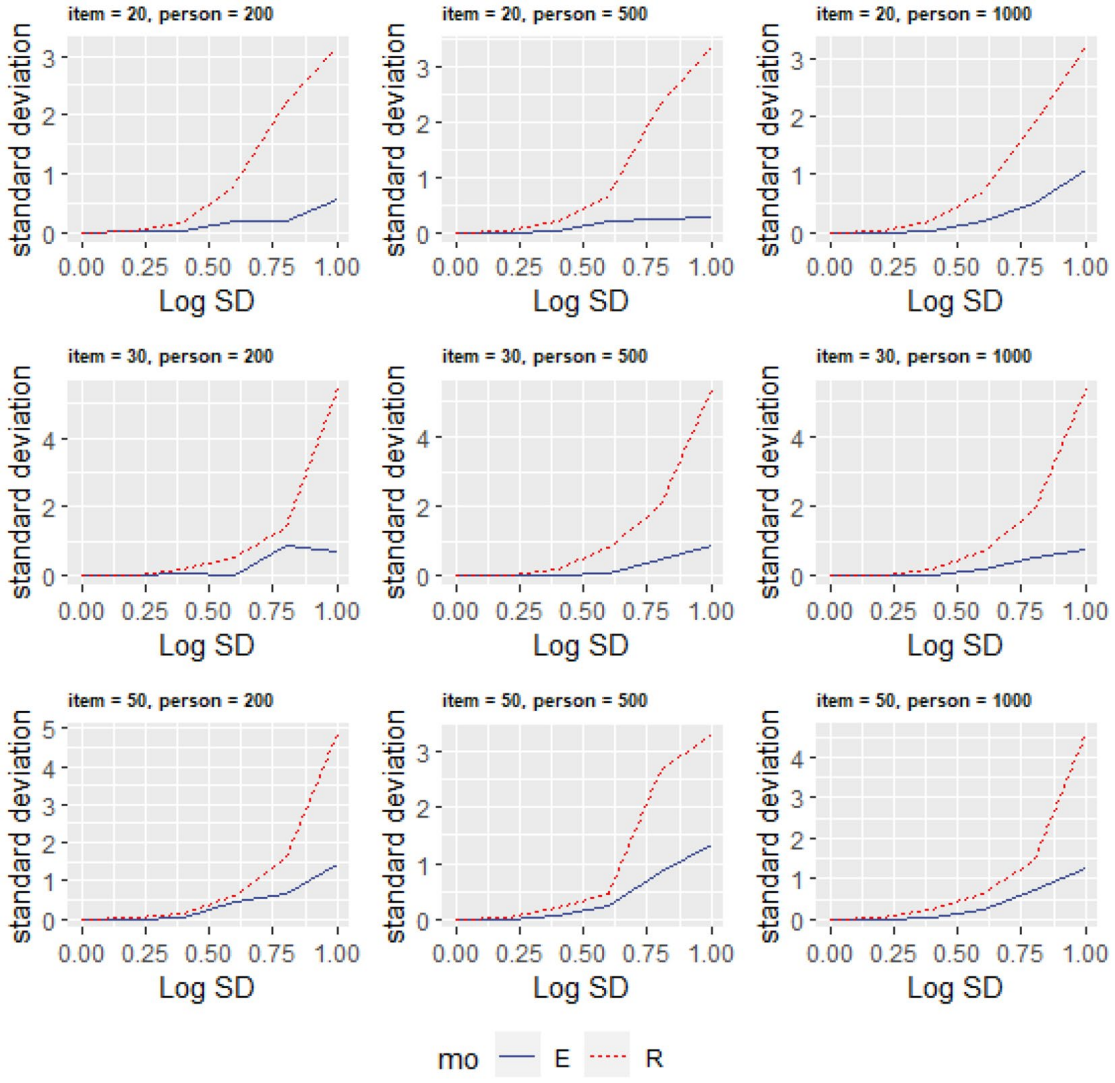
$\widehat{\varepsilon }$ Standard error and its estimated value under various conditions.

For the data generated by different models, we need to perform model selection. The difference between the IREM and classic IRT models is whether $\widehat{\varepsilon }$ changes among respondents. Figure 2 shows the standard deviation of $\widehat{\varepsilon }$ under different conditions. When the data is generated by an individual random effects model, ${S}_{\widehat{\varepsilon }}$, the estimated standard deviation of $\widehat{\varepsilon }$, is always smaller than the real standard deviation ${S}_{\varepsilon }$. When there is no individual random effect or the individual random effect is small, ${S}_{\widehat{\varepsilon }}$ is approximately equal to 0, and when the individual random effect is large enough, the standard deviation of $\widehat{\varepsilon }$ is considerably greater than zero. These simulation results provide evidence that the proposed model selection method is helpful for determining whether the data conforms to the classic IRT model or the individual random effect model. In other words, the model selection method helps determine whether the classic IRT model is sufficient or whether there are differences in within-person variance among respondents. In addition, individual random effects models can identify and estimate these deviations.

## Real data study

### Data and estimate

As example, we use the PRESUPP scale that came from the 2015 Program for International Student Assessment (PISA). Ten items are included in the scale, which ask respondents how frequently their child engaged in science-related learning activities at home when he or she was 10 years old, and then inquired about parents’ support for science learning in the middle childhood years from the following 10 aspects:

1. Watched TV programs about science,
2. Read books on scientific discoveries,
3. Watched, read or listened to science fiction,
4. Visited web sites about science topics,
5. Attended a science club,
6. Construction play, e.g. < lego bricks > 
7. Took apart technical devices,
8. Fixed broken objects or items, e.g. broken electronic toys,
9. Experimented with a science kit, electronics kit, or chemistry set, used a microscope or telescope,
10. Played computer games with a science content.

The response categories were “very often”, “regularly”, “sometimes”, “never” and had to be reverse-coded so that higher WLEs and higher difficulty correspond to higher levels of parental support. To adapt to the binary model, the responses “very often” and “regularly” are recorded as "1", which refers to higher frequency. The responses “sometimes” and “never” are recorded as "0", which refers to lower frequency.

This study uses the Croatian subset of the data, which contains N = 5220 participants’ responses on these 10 items. The mean proportion of “1” on each item is 0.02 to 0.61. To implement MCMC, we specify the priors, iterations and burn-in period as we described in Sect. "Parameter estimate". The computation took approximately 365 min for the individual random effects model and 48 min for 2PL on a computer with Intel(R) Core (TM) i7-11700 K CPU and 64G RAM. Trace plots show reasonable convergence of the sampler. In addition, we used S. P. Brooks’^32^ improved Gelman Rubin convergence statistics to detect possible non-convergence. We ran the model with three sets of random initial values. The scale reduction factor is smaller than 1.01 for all model parameters, suggesting that there are no signs of non-convergence. We implemented the model selection method described in Sect. "Simulation study". According to the results of the simulation study, when ${S}_{\widehat{\varepsilon }}$ is approximately equal to zero, the data fits a classic IRT model. ${S}_{\widehat{\varepsilon }}$ is considerably greater than zero and the data fits the individual random effects model. The obtained ${S}_{\widehat{\varepsilon }}$ is 0.21, which means that the within-person variances are different. The data is more consistent with the new model. Therefore, we move forward with the individual random effects model for the current application.

### Statement

This research involves the utilization of publicly available psychological measurement data from human participants. The dataset used in this study originates from Programme for International Student Assessment (PISA), which adheres to ethical guidelines and privacy policies. Throughout the research process, we have strictly followed the guidance and ethical principles provided by the relevant committee to ensure the protection of participants' privacy and rights.

In the original dataset, all personally identifiable information has been removed, and data is presented in an anonymized manner to safeguard the privacy of the participants. The analysis and interpretation of the data focus solely on overall trends and patterns without involving any content that could potentially identify individual participants.

### Results

#### Goodness-of-fit analyses

Model fit often uses model fitting indices: − 2 log-likelihood values (− 2LL), the Akaike's information criterion (AIC) and the Deviance information criterion (DIC). But AIC and DIC need the sample size to be much larger than the number of parameters^38^, so they are not suitable for our IRT model. Stan used the WAIC and the LOO for model comparison and selection because they were completely based on Bayesian theory and were theoretically superior to classic information-based model selection indicators. In the context of IRT model selection, Luo Yong^39^ studied the performance of the WAIC and the LOO on the dichotomously IRT model and found that they were superior to classic methods. Therefore, this study compares the fitness of the three models through model fitting indicators: − 2LL, WAIC and LOO.

Table 3 shows the model fitting indices of the three models. The results show that compared with the 1PL model and the 2PL model, the individual random effects model performs better on all the three fitting indices: -2LL, WAIC, and LOO.

**Table 3:** Relative fitting index of model.

Model	Fitting indices		
	− 2LL	WAIC	LOO
1PL	31,557.33	35,092.6	35,240.8
2PL	31,655.08	34,660.5	34,797.1
IREM	29,921.61	33,952.2	34,461.7

#### Comparison with the classic IRT model

We compare the estimated parameter results with the classic IRT model. The classic IRT model also uses the MCMC method for estimate and has the same priori as the individual random effects model.

**Figure 3:**
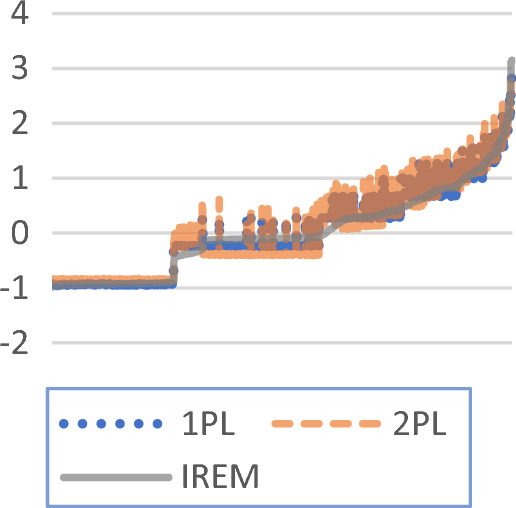
Estimated values of test parameters of different models.

The estimated values of the respondent’s ability of the three models are shown in Fig. 3. In general, the estimates of the three models are similar, and the correlation between the results of the individual random effects model and classic IRT models is 0.984 and 0.981. However, the estimate results of some particular respondents are different, and the difference comes from various parameter restrictions. The 2PL restriction has a constant within-person variance, and the 1PL has an additional restriction that all the items have a constant slope. The new model releases both these two restrictions.

**Figure 4:**
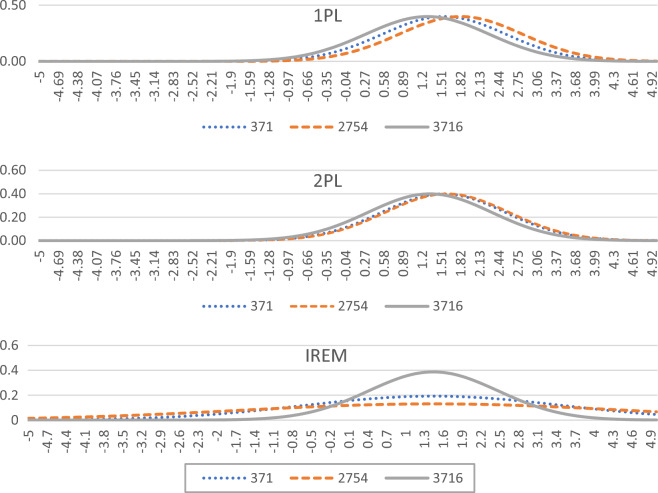
Estimate of parameters of different models and their distribution.

The new model not only estimates the position parameter information of the respondent’s ability but also estimates its scale information. For example, the abilities of respondents 371, 2754, and 3716 have the same abilities of the new model, which are all 1.45, but the option probabilities of the three respondents are quite different. The response matrix is shown in Table 4. The estimated values of 1PL for the three respondents' abilities are 1.56, 1.83, and 1.28; the estimated values of 2PL are 1.55, 1.61, and 1.33. The classic IRT model believes that this difference is caused by different abilities. The new model estimates different within-person variances based on the difference in option probabilities. If the parameter of within-person variances is introduced, the estimated abilities of the three respondents are shown in Fig. 4.

**Table 4:** Response matrix of three respondents.

ID	Item1	Item2	Item3	Item4	Item5	Item6	Item7	Item8	Item9	Item10
P371	1	1	0	1	0	1	0	1	0	1
P2754	1	1	1	1	1	1	0	0	0	1
P3716	0	0	1	0	0	1	1	1	0	1

Figure 4 shows that the classic IRT model is not sensitive to the differences in the response pattern of the respondents, and the differences in response pattern are manifested as small differences in the ability. In the new model, the three respondents have the same ability with different within-person variances, and the difference in response pattern comes from different within-person variances.

## Discussion

### Summary

From the perspective of the mixed effect location scale model, we relax the restriction on the magnitude of the within-person variances in the IRT model and introduce the scale (within-person variances) parameter to construct a new IRT model. The new model is more flexible, more realistic, and has some theoretical significance and practical value. The advantage of the parameter estimation accuracy of the new model is demonstrated through a simulation study, and finally, the new model is compared with the classic IRT model using a real data study. The main research findings are:

1. A Monte Carlo simulation study showed that the individual random effects model can obtain an unbiased estimate of the respondent’s ability, and its RMSE value is not larger than that of the classic IRT models. Moreover, when data is generated by the individual random effects model, the RMSE value of the individual random effects model is smaller than that of the classic IRT models, which suggests that the individual random effects model has better parameter estimation accuracy than the two-parameter model when there are differences in within-person variance.
2. When the data is generated by the individual random effect model, and the standard deviation of $\widehat{\varepsilon }$ is large enough, the estimate of the standard deviation of $\widehat{\varepsilon }$ can be used for model identification. As the individual random effect increases, the estimate of the standard deviation of $\widehat{\varepsilon }$ also increases.
3. The practical effects of the 1PL, 2PL, and IREM are compared using the 2015 PISA Parents’ Support for Science Learning Questionnaire in Middle Childhood. The fit indices show improvement, and they are sensitive to the differences in the response pattern.

### Limitation and possible applications

Although the 1PL and 2PL models are classic models and fit well in most cases, many studies have revealed that some respondents will deviate from the model, which implies that there are unexplainable differences among the respondents. We demonstrate the advantages of the individual random effects model through simulation studies. In addition, we present evidence of individual differences in real data. At the same time, in some other datasets we tested (e.g. other country’s subset of PRESUPP, the Neuroticism scales of the Eysenck questionnaire and a test of mathematics, the last two are reported in “Appendix A” of the supplement), we observe that differences in within-person variance also exist. However, it is worth noting that to ensure that the results of the research do not lose generality and to further advance related research in the future, the following aspects should be studied:

1. The new model can estimate the within-person variance of each respondent (the Pearson correlations between $\widehat{\varepsilon }$ and $\varepsilon$ in the simulation study ranged from 0.544, which increased as the number of items increased, to 0.692, providing evidence that the models are accurately implemented, as reported in “Appendix B” of the supplement). However, it requires too many items to get an accurate estimate of the within-person variances, which necessitates the introduction of polytomous response formats to the new model.
2. Estimating the within-person variance can help detect undesired forms of response behavior. For example, in psychometrics, there are inadequate responses and false responses, and an abnormal increase in the within-person variance may indicate that the respondent’s own condition is unstable or they do not respond seriously. However, the MCMC method used in this study underestimates the difference in the within-person variance and cannot accurately estimate its value, while the MCMC method takes a long time and is not conducive to practical application. It is necessary to further develop other parameter estimation methods for this purpose.
3. With the development of the IRT, researchers have proposed a large number of revised models, such as the four-parameter (4PL) model, which introduces lower asymptotic parameters (also known as guessing coefficients) and upper asymptotic parameters (also known as sleep coefficients) based on the classic 2PL model^40^. The Response-Time IRT Models consider the respondents’ response time and add the item response time parameters^41^. The decision tree model (IRTree models) consider the respondent’s preference response tendency for different positions^6^. These models all study the differences among respondents, and future research can focus on the differences and connections between the individual random effects models and these models.
4. Further research showed that when the distribution of respondents’ abilities was broader than the distribution of item difficulty, the advantage of IREM for the accuracy of respondents’ ability estimation was more evident (Some results are reported in “Appendix C” of the supplement). This seems to imply that respondents who deviated from item difficulty were more likely to be misestimated in ability if within-person variances were neglected. This necessitates further mathematical derivation and empirical research.

### Supplementary Information

Supplementary Information 1.

Supplementary Information 2.

Supplementary Information 3.

## Data Availability

The datasets utilized in this study comprise a combination of simulated generated data and publicly accessible data sourced from the Programme for International Student Assessment (PISA). The PISA data, integral to this research, can be freely obtained from the official PISA website (https://www.oecd.org/pisa/data/).

## Code availability

The code has been uploaded as an attachment only for the purpose of replicating results. In our subsequent research, we have made some improvements to the code to make it more adaptable and efficient. The latest code can be obtained from the corresponding author on reasonable request.
