# Supplementary material for: Individual random effects model for differences in trait distribution among respondents
Source: Sci Rep. 2024 May 25;14:12004. doi: 10.1038/s41598-024-62479-0 (PMC11128004; doi:10.1038/s41598-024-62479-0)
Supplement: Supplementary file 1 — Supplementary Information 1. [file 41598_2024_62479_MOESM1_ESM.docx]

Relative fitting index of model (a test of mathematics)

| Model | Fitting indices | | | | |
| --- | --- | --- | --- | --- | --- |
|  | -2LL |  | WAIC |  | LOO |
| 1PL | 8958.155 |  | 9706.1 |  | 9719.0 |
| 2PL | 8814.848 |  | 9307.4 |  | 9325.1 |
| IREM | 8670.657 |  | 9256.7 |  | 9301.5 |

$S_{\hat{\sigma_{P}}}=$0.190

The computation took approximately 938 seconds for the individual random effects model and 470 seconds for 2PL

Relative fitting index of model (EPQ N)

| Model | Fitting indices | | | | |
| --- | --- | --- | --- | --- | --- |
|  | -2LL |  | WAIC |  | LOO |
| 1PL | 67882.52 |  | 70655.1 |  | 70678.1 |
| 2PL | 67551.11 |  | 69905.8 |  | 69929.8 |
| IREM | 66873.99 |  | 69790.6 |  | 69854.3 |

$$S_{\hat{\sigma_{P}}}= 0.060$$

The computation took approximately 6875 seconds for the individual random effects model and 3241 seconds for 2PL
