# Supplementary material for: Individual random effects model for differences in trait distribution among respondents
Source: Sci Rep. 2024 May 25;14:12004. doi: 10.1038/s41598-024-62479-0 (PMC11128004; doi:10.1038/s41598-024-62479-0)
Supplement: Supplementary file 2 — Supplementary Information 2. [file 41598_2024_62479_MOESM2_ESM.docx]

Pearson correlations between $\hat{\sigma_{P}}$ and $\sigma_{P}$（Average of 30 cycles）

| Items | N = 20 | N = 30 | N = 50 |
| --- | --- | --- | --- |
| 200 | 0.581 | 0.604 | 0.696 |
| 500 | 0.549 | 0.613 | 0.704 |
| 1000 | 0.544 | 0.606 | 0.692 |
