# Supplementary material for: Individual random effects model for differences in trait distribution among respondents
Source: Sci Rep. 2024 May 25;14:12004. doi: 10.1038/s41598-024-62479-0 (PMC11128004; doi:10.1038/s41598-024-62479-0)
Supplement: Supplementary file 3 — Supplementary Information 3. [file 41598_2024_62479_MOESM3_ESM.docx]

RMSE values of potential trait levels of respondents under various conditions generated by the new model

| Number of Respondents | Items | 1PL | 2PL | IREM |
| --- | --- | --- | --- | --- |
| 200 | 20 | 1.06 | 1.03 | 0.94 |
|  | 50 | 0.99 | 0.97 | 0.81 |

the abilities are evenly distributed
